# Structural Changes in Semi-Crystalline Ethylene-Based Ionomers During the Heating Process

**DOI:** 10.3390/polym17010037

**Published:** 2024-12-27

**Authors:** Shunsuke Murayama, Go Matsuba

**Affiliations:** Graduate School of Organic Materials Science, Yamagata University, 4-3-16 Jonan, Yonezawa 992-8510, Japan; t221170d@st.yamagata-u.ac.jp

**Keywords:** ionomer, polyethylene, crystallization, X-ray scattering measurements, semi-crystalline polymers, ethylene-based polymers, thermal behavior, microstructural evolution, differential scanning calorimetry (DSC), wide-angle X-ray scattering (WAXS), small-angle X-ray scattering (SAXS), polyethylene crystals, ionic aggregates

## Abstract

The structural behavior of ionic aggregates in semi-crystalline ethylene-based ionomers during heating was studied to understand the influence of different ionic groups on their properties. The ionomers were synthesized by neutralizing carboxylic acid groups with Zn and Na ions at varying ratios. Differential scanning calorimetry (DSC) revealed two distinct endothermic peaks, with the melting point being largely unaffected by the Zn/Na ion ratio. However, the melting enthalpy of *T*_*i*_ is maximum at Na/Zn ratios of 3:7 and 5:5, suggesting that crystallites preferentially grow in the presence of both ions are present. In situ wide-angle X-ray scattering (WAXS) identified temperature-dependent transitions, where monoclinic crystals melted near *T*_*i*_ and recrystallized into orthorhombic forms, which subsequently melted as the temperature approached *T*_*m*_. Small-angle X-ray scattering (SAXS) analysis, employing the Yarusso–Cooper equation, indicated a temporary expansion of ionic aggregates near *T*_*i*_, followed by further expansion near *T*_*m*_. These expansions were attributed to the melting of polyethylene crystals, which were found to compress the aggregates under normal conditions. These findings provide insights into the relationship between ionic group composition, microstructural evolution, and thermal behavior in ethylene-based ionomers, with implications for their application in temperature-sensitive environments.

## 1. Introduction

An ionomer is a generic term for polymers containing small amounts of ionic groups either in the side chains or the main chain of a base polymer. Among these, ethylene-based ionomer resins, which consist of ethylene as the hydrophobic polymer backbone and a small fraction of carboxyl groups introduced as ionic groups, are notable for their excellent toughness, transparency, and abrasion resistance. These properties make them highly versatile for applications such as golf ball covers and food packaging materials.

Ethylene-based ionomers are synthesized from ethylene-methacrylic acid copolymers (E/MAA) by partially neutralizing the carboxyl groups with metal cations, such as zinc or sodium. This process results in the formation of “ionic aggregates” at the nanoscale, creating a complex three-phase structure composed of crystalline and amorphous phases of polyethylene, along with an amorphous phase arising from the aggregated neutralized ions [1].

Due to these intricate physical properties, there has been extensive research on the relationship between structure and properties, employing techniques such as X-ray scattering [2,3,4,5], transmission electron microscopy [6], X-ray spectroscopy [7,8], atomic force microscopy [9,10], and melt viscoelasticity measurements [11,12]. Miwa et al., for instance, investigated the chain dynamics of ionomers, focusing on glass-transition temperature behavior [13,14].

Thermal analysis has revealed that EMAA copolymers and their ionomers exhibit two distinct endothermic peaks, approximately at 50 °C and 90 °C, corresponding to the melting temperature (*T*_*m*_) of polyethylene [15,16]. It is further established that the peak temperature (*T*_*i*_) and the enthalpy of the endothermic peak are influenced by heat treatment conditions and the degree of ionic neutralization [17,18]. However, the underlying cause of the observed *T*_*i*_ peak remains unclear.

Especially, crystallization processes greatly depend on the ionic neutralization ratio and/or ionic species. For example, in the case of mixtures of Zn and Na ions, when crystallinity increased the ratio of Zn:Na = 7:3 and 5:5, then the tensile strength and impact resilience improved [5]. Then, the influences of the ionic species and mixtures on crystallization have not been clarified.

The aim of this study is to elucidate the structural changes occurring in crystalline ethylene-based ionomers during temperature elevation. This was achieved through thermal measurements in conjunction with in situ small- and wide-angle X-ray scattering (SAXS and WAXS) analyses.

## 2. Materials and Methods

### 2.1. Materials

Ethylene-co-methacrylic acid (EMAA), a random copolymer of ethylene and methacrylic acid, was sourced from Mitsui Dow Polychemical Co., Ltd. (Tokyo, Japan) The experimental samples included “EMAA-Zn”, with 59% zinc ion neutralization, and “EMAA-Na”, with 54% sodium ion neutralization. In addition to these, bimetallic mixtures of EMAA-Zn and EMAA-Na were prepared at weight ratios of 10:0, 7:3, 5:5, 3:7, and 0:10 using an extruder at 200 °C for 5 min. The specific conditions for each blended sample are detailed in Table 1.

Prior to experimentation, the sample films were melted at 150 °C and rapidly cooled to a thickness of 100 μm using a Mini Test Press 10 (Toyo Seiki Co., Ltd., Tokyo, Japan). The samples were then isothermally annealed under various temperature conditions in a vacuum oven to prevent oxidation and degradation until just before the experiments.

### 2.2. Thermal Analysis

Differential scanning calorimetry (DSC) measurements were conducted using a DSC-20 instrument (TA Instruments, New Castle, DE, USA) to evaluate the melting behavior of polyethylene crystals. The measurements were performed within a temperature range of 30 °C to 120 °C at a heating rate of 10 °C/min. Approximately 4 mg of each sample was used for the DSC measurements. Since the polyethylene content varied across the samples due to the incorporation of Zn and/or Na ions in the ionomers, the melting enthalpy was normalized to the weight of polyethylene in each sample. This normalization was performed using estimated values derived from the DSC measurements.

### 2.3. X-Ray Scattering Measurements

Small-angle X-ray scattering (SAXS) and wide-angle X-ray scattering (WAXS) measurements were performed at the BL-6A beamline of the Photon Factory, High Energy Accelerator Research Organization (Tsukuba, Japan), with a wavelength of 0.15 nm [19,20]. The respective camera lengths for SAXS and WAXS were 1400 mm and 240 mm, while the detectors used were PILATUS 3 1M and PILATUS 100K (DECTRIS, Baden, Switzerland). The scattering vector, *q*, range (*q* = 4*π*sin*θ*/*λ*, where 2*θ* is the scattering angle and *λ* is the wavelength) extended from 0.1 to 6.5 nm^−1^ for SAXS and from 7 to 19 nm^−1^ for WAXS. Sample films, approximately 100 μm thick, were sandwiched between two polyether ether ketone (PEEK) films and mounted on a Linkam 10,002 L hot stage (Linkam Scientific Instruments, Redhill, UK) for precise temperature control [21,22]. In situ SAXS and WAXS measurements were conducted within a temperature range of 30 °C to 120 °C, with 2D images captured every 6 s. Data processing, including contrast adjustments of the 2D patterns and the generation of 1D profiles, was performed using FIT2D software (version 12.077, European Synchrotron Radiation Facility, Grenoble, France).

## 3. Results and Discussion

### 3.1. DSC Measurements

**Figure 1:**
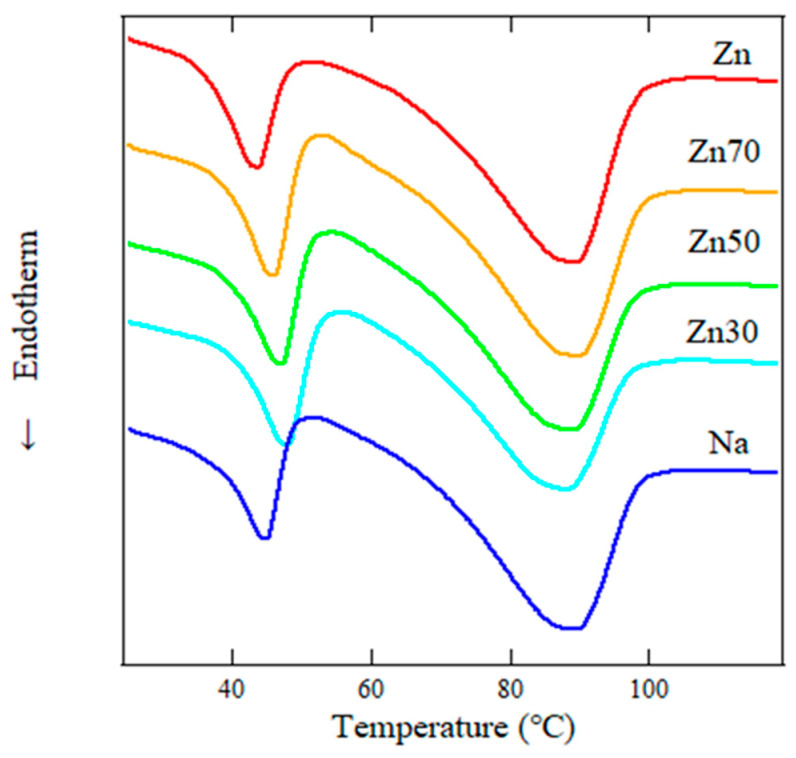
Effects of Na and Zn ion mixing on the thermal properties of ionomers. DSC thermograms illustrate the melting behavior of annealed samples during the first heating cycle, measured at a heating rate of 10 °C/min, in the temperature range of 30 °C to 120 °C.

Figure 1 displays the DSC thermograms obtained during the first heating cycle at a rate of 10 °C/min. The samples were annealed for one week at 23 °C (room temperature) in a vacuum. Two distinct endothermic peaks were observed for all samples.

The peak observed around 90 °C corresponds to the melting point (*T*_*m*_) of polyethylene crystals. The associated enthalpy change (Δ*H*_*Tm*_) was evaluated and found to strongly depend on the ratio of Na and Zn ions. The endothermic peak around 40 °C was identified as the characteristic peak (*T*_*i*_) of EMAA-type ionomers, which is attributed to the melting of small crystallites. This indicates enhanced crystallite growth during the one-week annealing period in the presence of both ions [17,23,24]. The enthalpy change associated with *T*_*i*_ (Δ*H*_*Ti*_) was also evaluated. These values for *T*_*i*_, Δ*H*_*Ti*_, *T*_*m*_, and Δ*H*_*Tm*_ are summarized in Table 2.

The values of *T*_*i*_ and Δ*H*_*Ti*_ were strongly dependent on the mixing ratio of Na and Zn ions. Notably, the Zn30 and Zn50 samples exhibited significantly larger *T*_*i*_ and Δ*H*_*Ti*_, suggesting the formation of larger crystallites. Conversely, the *T*_*m*_ and Δ*H*_*Tm*_ of Zn30 and Zn50 were markedly smaller than those of the other samples. Since *T*_*m*_ represents crystals formed during rapid cooling in sample preparation, while *T*_*i*_ reflects crystals that grow over days, the differences in crystallization during cooling were further analyzed [25,26].

**Figure 2:**
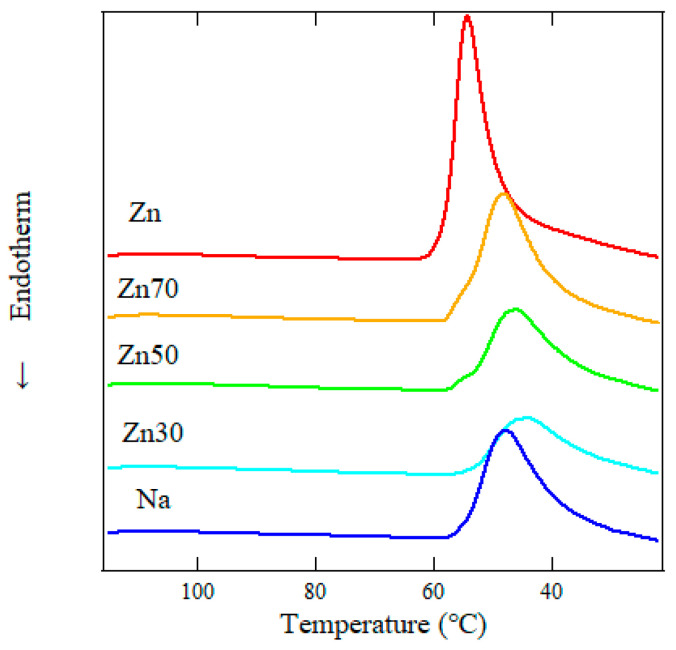
Differential crystallization behavior of ionomers influenced by mixing metal cations. DSC measurements illustrate the melting process at 120 °C for 5 min, followed by cooling from 120 °C to 30 °C at a cooling rate of 10 °C/min.

Figure 2 shows the DSC thermograms recorded during the cooling process, and Table 3 summarizes the corresponding crystallization temperatures and enthalpies. These results reveal that Zn30 and Zn50 ionomers crystallized less readily than the other samples. The significantly lower Δ*H*_*Tm*_ suggests reduced crystallization during cooling. In contrast, the higher Δ*H*_*Ti*_ indicates that crystals formed more readily during the extended annealing period, compensating for the limited crystallization during cooling.

### 3.2. Crystallinity from X-Ray Measurements

**Figure 3:**
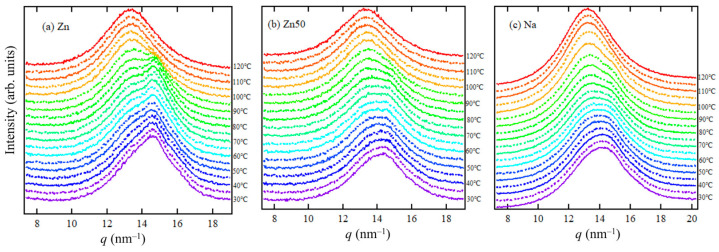
In situ WAXS profiles of Zn (**a**), Zn50 (**b**), and Na (**c**) samples at various temperatures during heating. The measurements were conducted at a heating rate of 10 °C/min, starting from 30 °C, to examine transitions between crystalline and amorphous states. The baseline of each profile has been shifted vertically to prevent overlap.

Figure 3a–c illustrates the temperature-dependent WAXS measurements during the heating process for Zn, Zn50, and Na samples. In Figure 3a, the polyethylene orthorhombic crystal diffraction peaks, specifically the (110) and (200) peaks at *q*-values of 14.7 and 16.0 nm^−1^, respectively, are observed for the Zn sample between 60 °C and 100 °C. Below 50 °C, these peaks appear ambiguous, but they become more prominent above *T*_*i*_. This indicates that the orthorhombic crystal begins to grow around *T*_*i*_.

**Figure 4:**
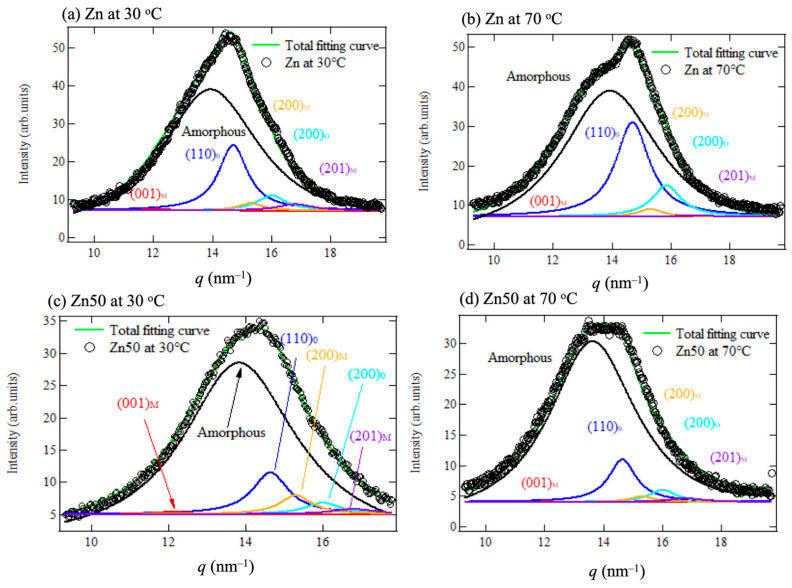
(**a**–**d**) Peak fitting of the WAXS profile for the Zn sample at 30 °C (**a**) and at 70 °C (**b**) and Zn50 sample at 30 °C (**c**) and at 70 °C (**d**). The black diamonds represent the experimental scattering profile, while the green line corresponds to the overall fitting curve. The blue and light blue lines denote the orthorhombic PE crystal diffraction peaks (110)_*O*_ and (200)_*O*_, respectively. The red, orange, and purple lines represent the monoclinic PE crystal diffraction peaks (001)_*M*_, (200)_*M*_, and (201)_*M*_, respectively.

For a more detailed quantitative evaluation of the WAXS profiles, curve fitting analysis was performed in the *q*-range of 10–18 nm^−1^. This analysis accounted not only for the orthorhombic polyethylene (PE) crystal peaks but also for the monoclinic PE crystals, as previously noted in the literature [27,28]. Asano et al. demonstrated that ethylene-based ionomers exhibit crystalline phases that include monoclinic crystals alongside orthorhombic ones [27]. Consequently, each crystalline phase was individually separated and evaluated. The results of the curve fitting, which distinguished contributions from the amorphous halo, orthorhombic, and monoclinic crystal diffraction, are presented in Figure 4a–d for the Zn samples and Zn50 samples at 30 °C and 70 °C.

Subsequently, the crystallinity of the samples was evaluated at various temperatures. The ratio of the crystalline region, *R*_*c*_(*T*), is defined by Equation (1):

(1) $$R_{c}\left(T\right)=\frac{I_{cry,ortho}\left(T\right)+I_{cry,mono}\left(T\right)}{I_{cry,ortho}\left(T\right)+I_{cry,mono}\left(T\right)+I_{amo}(T)}$$

**Figure 5:**
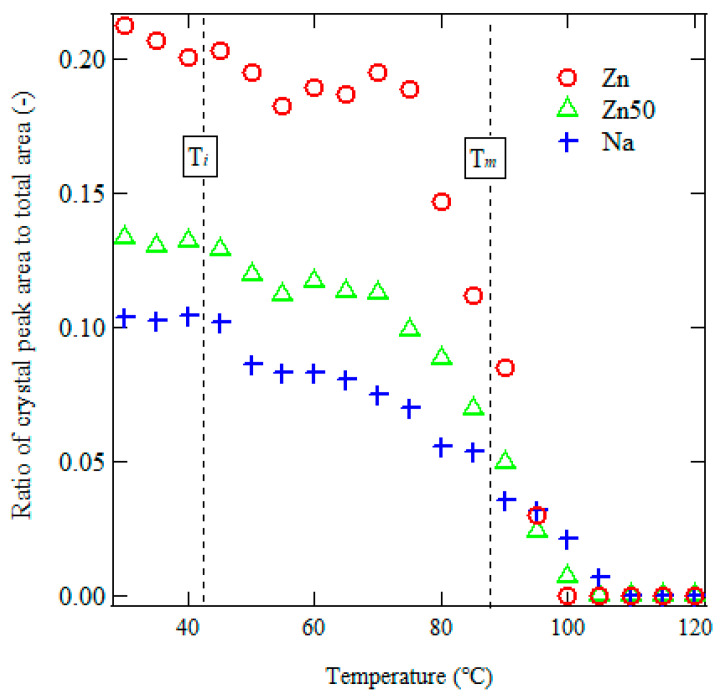
Temperature dependence of *R*_*c*_(*T*) for Zn, Zn50, and Na samples derived from in situ WAXS profiles during heating (as shown in Figure 3). The crystallinity ratio, *R*_*c*_(*T*), is calculated using Equation (1), highlighting changes in crystallinity during heating, including the effects of melting, recrystallization, and crystalline phase transitions.

Here, *I*_cry,ortho_(*T*), *I*_cry,mono_(*T*), and *I*_amo_(*T*) represent the areas of the orthorhombic, monoclinic polyethylene (PE) crystalline regions, and amorphous regions in the XRD profiles, respectively. Figure 5 shows the temperature dependence of *R*_*c*_(*T*) in the various samples.

The *R*_*c*_(*T*) decreased from 30 °C to *T*_*i*_, consistent with the endothermic peak observed in the DSC thermograms. Upon further heating, *R*_*c*_(*T*) increased between *T*_*i*_ and *T*_*m*_ in all samples, indicating recrystallization above *T*_*i*_, likely due to the melting of smaller crystallites. Above *T*_*m*_, *R*_*c*_(*T*) decreased with increasing temperature, reflecting the melting of PE crystals.

However, the crystallinity values derived from Δ*H*_*Ti*_ and/or Δ*H*_*Tm*_ in the DSC results do not consistently align with the behavior of *R*_*c*_(*T*) at 30 °C shown in Figure 5. This discrepancy arises because Δ*H*_*Ti*_ and Δ*H*_*Tm*_ include contributions from both orthorhombic and monoclinic crystals, whereas *R*_*c*_(*T*) calculated from in situ WAXS measurements considers only the ratio of crystalline to amorphous regions.

Furthermore, during DSC measurements, melting and recrystallization processes occurring during heating can obscure the precise determination of crystallinity using a simple area ratio alone [29].

For a more detailed analysis of the temperature dependence, we evaluated the ratios of orthorhombic crystals, *R*_*o*_(*T*), and monoclinic crystals, *R*_*m*_(*T*), using Equations (2) and (3) based on the in situ WAXS measurements:

(2) $$R_{o}\left(T\right)=\frac{I_{cry,ortho}\left(T\right)}{I_{cry,ortho}\left(T\right)+I_{cry,mono}\left(T\right)+I_{amo}(T)}$$

(3) $$R_{m}\left(T\right)=\frac{I_{cry,mono}\left(T\right)}{I_{cry,ortho}\left(T\right)+I_{cry,mono}\left(T\right)+I_{amo}(T)}$$

**Figure 6:**
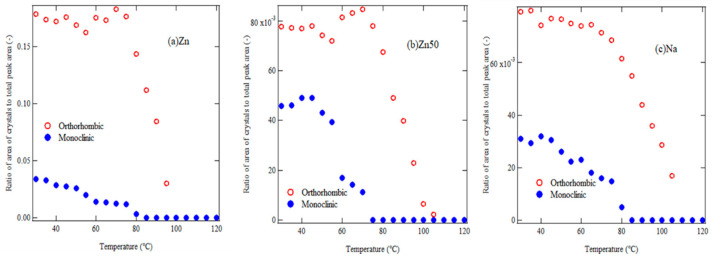
Temperature dependence of the area ratio of orthorhombic (*R*_*o*_(*T*)) and monoclinic (*R*_*m*_(*T*)) crystals for Zn (**a**), Zn50 (**b**), and Na (**c**) samples. Red open circles represent the orthorhombic crystal ratio, and blue, filled circles represent the monoclinic crystal ratio.

Figure 6a–c show the temperature dependence of the orthorhombic and monoclinic crystal components. The amount of monoclinic crystals decreases until *T*_*i*_, while the amount of orthorhombic crystals increases slightly. During the melting of monoclinic crystals, orthorhombic crystals grow. Between *T*_*i*_ and *T*_*m*_, the monoclinic component remains constant during heating and then decreases around *T*_*m*_. These results suggest that small monoclinic crystallites melt around *T*_*i*_, while orthorhombic PE crystals grow during the recrystallization process above *T*_*i*_. Furthermore, since orthorhombic crystals melt near the melting point *T*_*m*_, the endothermic peak at *T*_*m*_ can be attributed to the melting of orthorhombic crystals. For a more detailed analysis, we next discuss the ionic correlation from SAXS measurements.

### 3.3. Ionic Correlation from SAXS Measurements

**Figure 7:**
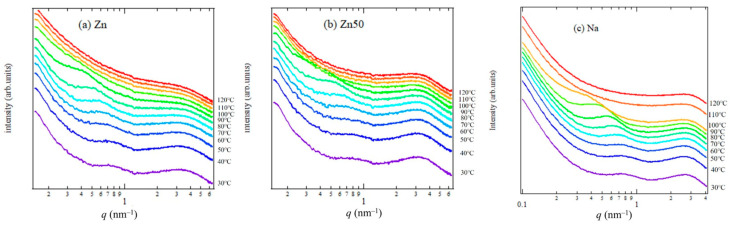
In situ SAXS profiles at various temperature conditions during the heating of Zn (**a**), Zn50 (**b**), and Na (**c**) samples. The samples were heated at a rate of 10 °C/min from their crystalline/amorphous states starting at 30 °C. The baseline of each profile has been shifted vertically for clarity and to prevent overlap.

Figure 7a–c shows the in situ SAXS profiles during the heating process of the annealed samples. Two peaks were observed in all samples: one between *q* = 0.3 nm^−1^ and 1.0 nm^−1^ and the other between *q* = 2.0 nm^−1^ and 4.0 nm^−1^ from 30 to 80 °C.

**Figure 8:**
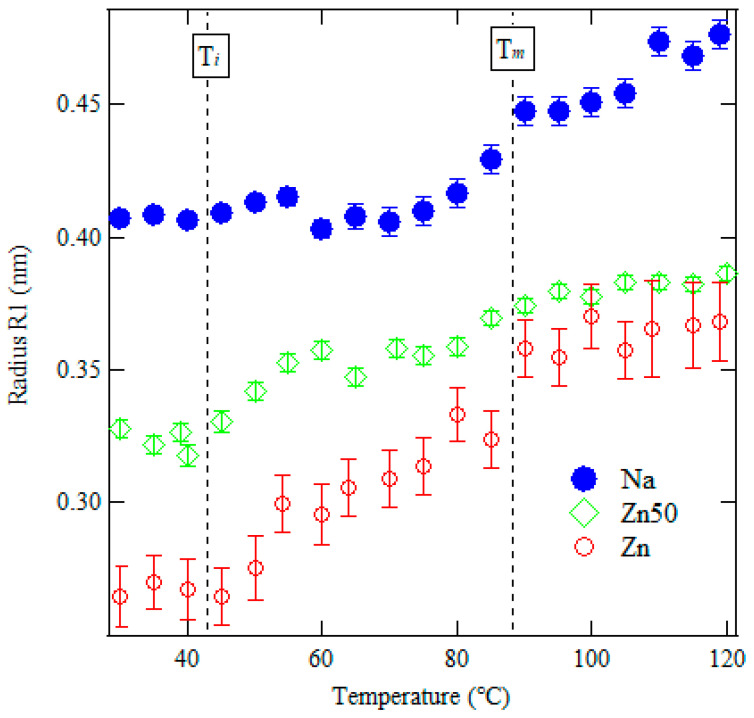
Temperature dependence of the radius of the ion aggregate, R1, for various blended samples (Zn, Zn50, and Na). The error bars are included for the Na and Zn50 samples, as indicated by the markers.

The peak at *q* = 0.3−1.0 nm^−1^ shifted toward the small-angle region during heating, indicating an increase in the correlation length between crystallites as the temperature rose. Upon further heating, this peak disappeared due to the melting of crystals. In a previous study, we discussed the crystal growth and melting processes in various EMAA ionomers. The second peak at *q* = 2.0−4.0 nm^−1^ corresponds to the correlation between the ion aggregates. The correlation length of the ion aggregates increased with temperature due to thermal expansion [23]. To investigate the temperature dependence of the ion clusters, we applied the Yarusso–Cooper equation [2], as shown in Equation (4):

(4) $$I\left(q\right)=I_{e}\left(q\right)\cdot V\frac{1}{v_{p}}v_{1}^{2}\rho_{1}^{2}\Phi^{2}(qR_{1})\frac{1}{1+(8v_{ca}/v_{p})\varepsilon \Phi (2qR_{ca})}\;\Phi \left(x\right)=\frac{3\left(\sin x-x\cos x\right)}{x^{3}}$$

where *R*_1_ is the radius of the ionic aggregate, *R*_*ca*_ is the motion-bound region of the ionic aggregate, and *I*_*e*_(*q*) is the scattering function from one electron. *v*_*p*_, *v*_1_, *v*_*ca*_, and *ρ*_1_ are the mean volume of the ion aggregates, the volume of the ionic aggregate nucleus, the volume of the kinetically bound region, and the density fluctuation, respectively. Figure 8 shows the temperature dependence of the radius of the ionic aggregates. In Na and Zn50 samples, the error bar is so small within each marker.

From 30 to 80 °C, the *R*_1_ decreases with increasing Zn ion concentration, suggesting that the radius of the ion aggregates increases with Na content in the mixed ionomers. We predicted that the strong aggregation of Zn50 and Zn30 ionic aggregates will result in less crystallization during cooling due to the restricted molecular chains. The ionic radii of Zn and Na ions, with coordination numbers of 4 and 6, are 0.060 nm and 0.102 nm, respectively [30,31]. However, *R*_1_ is significantly larger than the ionic radius of these ions, indicating that the ionic aggregates consist of 3 or 4 Na/Zn ions and carboxyl groups. Before *T*_*i*_, *R*_1_ remains constant for both samples. During heating, especially above *T*_*i*_, *R*_1_ begins to increase with temperature due to thermal expansion. This suggests a correlation between the melting of crystals and the increase in the radius of ionic aggregates. The presence of crystalline regions appears to compress the ionic aggregates. Above 80 °C, the error bars for the Zn sample become large due to the high electron density of Zn ions, which weakens the intensity of the peak at *q* = 4.0 nm^−1^. Despite this, no significant expansion of *R*_1_ is observed after the melting of PE crystals. This indicates that the expansion of ionic aggregates is not driven by thermal motion in the temperature range up to 120 °C. Furthermore, Zn50 and Zn30 samples are less likely to crystallize upon cooling in Figure 2, while the transition enthalpy of *T*_i_ was higher than Zn or Na sample in Table 2. Tachino et al. suggested that ionomers with complex ions form conjugated binary metal salts in the ionic aggregates, which leads to the formation of more cohesive aggregates [32]. This suggests that the growth of monoclinic crystals was attributed to heat treatment at room temperature for one week. Then, the strong aggregation of mixed ionic aggregates would prevent crystallization during cooling due to the restricted molecular chains.

## 4. Conclusions

The ionomer-specific endothermic peak (*T*_i_) was found to shift to a higher temperature and show increased enthalpy with the inclusion of two types of metal ions, suggesting enhanced crystallite growth. The crystallites in ethylene-based ionomers are believed to be both monoclinic and orthorhombic polyethylene crystals. The growth of monoclinic crystals was attributed to heat treatment at room temperature for one week. These monoclinic crystals were preferentially melted during the heating process, with recrystallization occurring as the temperature increased. This indicates that the monoclinic crystals grew during melting, leading to a temporary increase in the number of crystals. Furthermore, during the heating process, the ionic aggregates expanded, as evidenced by an increase in the aggregate radius, which was linked to the melting of the crystals. This expansion was caused by the presence of the crystals, which exerted pressure on the ionic aggregates.

## Figures and Tables

**Table 1:** Mass ratio of each ionomer sample mixing EMAA-Zn and EMAA-Na.

Sample	EMAA-Zn (wt%)	EMAA-Na (wt%)
Zn	100	0
Zn70	70	30
Zn50	50	50
Zn30	30	70
Na	0	100

**Table 2:** Transition endothermic peak (*T*_i_), its enthalpy change (Δ*H T*_i_), the melting of polyethylene crystals (*T*_m_), and its enthalpy change (Δ*H T*_m_) obtained by DSC measurements.

Sample	*T*_i_ (°C)	Δ*H T*_i_ (J/g)	*T_m_* (°C)	ΔH *T_m_* (J/g)
Zn	43.5	8.45	89.2	45.3
Zn70	45.9	8.60	89.6	36.0
Zn50	47.0	9.56	88.7	31.8
Zn30	47.9	9.53	87.8	28.6
Na	44.7	7.49	89.2	35.4

**Table 3:** Transition exothermic peak, *T*_c_, and its enthalpy change, Δ*H T*_c_, obtained by DSC measurements.

Sample	*T_c_* (°C)	ΔH *T_c_* (J/g)
Zn	54.3	45.5
Zn70	48.1	33.6
Zn50	45.9	24.1
Zn30	44.0	17.9
Na	47.6	28.8

## Data Availability

Raw data were generated at Yamagata University. Derived data supporting the findings of this study are available from the corresponding author, S.M. or G.M., on request.
